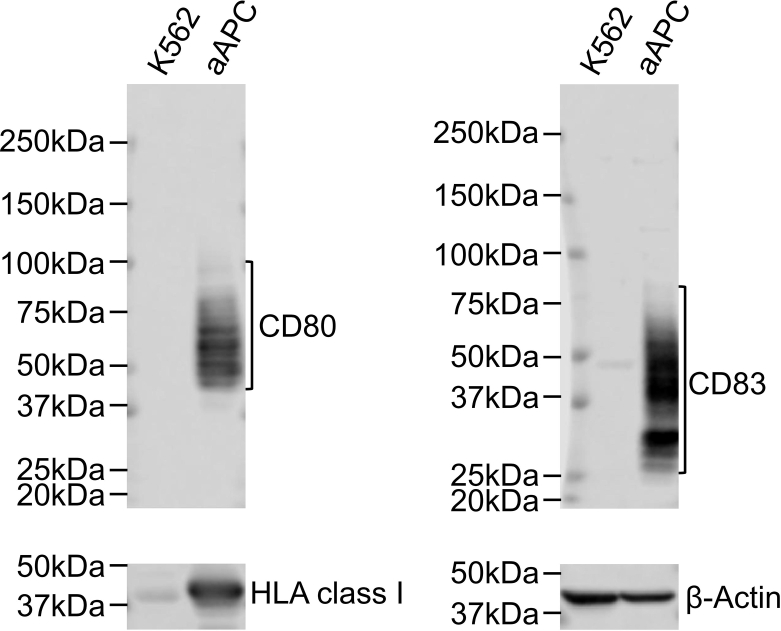# MHC class I trafficking signal improves induction of cytotoxic T lymphocyte using artificial antigen presenting cells

**DOI:** 10.1016/j.bbrep.2025.101946

**Published:** 2025-02-19

**Authors:** Kenta Sasaki, Kenji Murata, Tomoyuki Minowa, Naoki Shijubou, Hiroki Kobayashi, Munehide Nakatsugawa, Aiko Murai, Yuka Mizue, Terufumi Kubo, Takayuki Kanaseki, Tomohide Tsukahara, Hisashi Uhara, Akemi Ishida-Yamamoto, Yoshihiko Hirohashi, Toshihiko Torigoe

**Affiliations:** a Department of Pathology, Sapporo Medical University School of Medicine, Sapporo, Hokkaido, 060-8556, Japan; b Department of Dermatology, Sapporo Medical University School of Medicine, Sapporo, Hokkaido, 060-8556, Japan; c Department of Respiratory Medicine and Allergology, Sapporo Medical University School of Medicine, Sapporo, Hokkaido, 060-8556, Japan; d Department of Dermatology, Asahikawa Medical University School of Medicine, Asahikawa, Hokkaido, 078-8510, Japan; e Department of Pathology, Tokyo Medical University Hachioji Medical Center, Hachioji, Tokyo, 193-0998, Japan

**Keywords:** Artificial antigen-presenting cells (aAPC), Erythroleukemia cell line K562, MHC class I trafficking signal (MITD)

## Abstract

Inducing antigen peptide-specific cytotoxic T cells is challenging, partly due to the difficulty of maintaining the quality of antigen-presenting cells, such as dendritic cells. Consequently, artificial antigen-presenting cells (aAPCs) derived from the erythroleukemia cell line K562 have been employed for T cell stimulation. K562-based aAPCs can be utilized for both non-specific and antigen-specific T cell stimulation. Antigen peptide-pulsed aAPCs are commonly used to stimulate T cells with known specific antigenic peptides, which require identifying antigenic peptides from cognate antigen proteins. Therefore, antigen gene-overexpressing aAPCs might be useful for detecting unknown antigenic peptides. In this study, we evaluated the efficacy of cytotoxic T lymphocyte (CTL) induction using antigen gene-overexpressing aAPCs. To enhance antigen presentation efficiency, we assessed the signal peptide (SP) fused with MHC class I trafficking signal (MITD) sequences (SP-MITD). SP-MITD, fused with epitopes from the neoantigen AKF9 and the viral antigen CMVpp65, was transduced into aAPCs. We compared the CTL induction ability of peptide-pulsed aAPCs, only mini-gene-overexpressing aAPCs [SP-MITD (−)], and mini-gene fused with SP-MITD overexpressing aAPCs [SP-MITD (+)]. The SP-MITD (+) gene-overexpressing aAPCs exhibited the highest CTL induction efficiency compared to both peptide-pulsed and SP-MITD (−) gene-overexpressing aAPCs. These findings suggest that antigen gene-fused with SP-MITD transduced aAPCs are highly effective for inducing CTLs specific to both known and unknown antigenic peptides.

## Highlights

• SP-MITD fused protein is localized partially on the cells surface.
• SP-MITD (+) construct was overexpressed in aAPCs.
• CTL induction was compared using SP-MITD (+), peptide pulsed, SP-MITD (−) aAPCs.
• SP-MITD (+) overexpressed aAPCs showed highest CTL induction.

## 1 Introduction

Antigen-specific cytotoxic T lymphocytes (CTLs) play an essential role in cancer immune surveillance [1]. CTLs recognize antigenic peptides presented by major histocompatibility complex (MHC) class I molecules through the T cell receptor (TCR). Although the theoretical diversity of TCRs can be estimated to be around 10^12, the actual diversity is at least 2.5 × 10^7 [2]. Therefore, detecting an epitope-specific TCR is challenging. Antigen-specific CTLs can be propagated *in vitro* with appropriate stimulation [3]. Antigen-presenting cells (APCs) are used to stimulate T cells, and mature dendritic cells (DCs) are employed as APCs to generate antigen-specific CTLs due to their high expression levels of co-stimulatory molecules [[4], [5], [6]]. However, insufficient cell numbers and heterogeneity of monocyte-derived dendritic cells are obstacles to using DCs as APCs. To overcome these issues, artificial APCs (aAPCs) that express co-stimulatory molecules are suggested as alternatives [7].

An erythroleukemia cell line, K562 cells, is used as a human cell-based aAPC because K562 cells do not express MHC class I and co-stimulatory molecules but do express ICAM-1 and LFA-3 molecules, which play an important role in forming the immunological synapse [8], and K562 cells overexpressed co-stimulatory molecules or K562 cells conjugated with antibodies for co-stimulatory molecules could generate antigenic peptide specific CTLs. Therefore, K562 cells are commonly used as aAPCs for both MHC class I and MHC class II stimulation [9,10]. For stimulation of CD8^+^ T cells by aAPCs, aAPCs pulsed with epitope peptides are used. MHC class I presents antigenic peptides that are endogenously expressed and processed. MHC class I ligand antigenic peptides can be predicted by the anchor motif for binding MHC class I; however, very few candidate peptides are truly presented on MHC class I endogenously. Thus, an aAPC system that endogenously expresses antigens is essential.

In this study, we compared the CTL induction efficacy using peptide-pulsed aAPCs and aAPCs endogenously expressing antigen genes. We found that aAPCs transduced with antigen genes fused with a signal peptide (SP) and MHC class I trafficking signal (MITD) showed the highest CTL induction efficacy. These findings indicate that aAPCs expressing antigens with SP-MITD can induce CTLs efficiently.

## 2 Materials and methods

### 2.1 Cell lines, cell culture, plasmids

K562 and 293T cells were obtained from the American Type Culture Collection (Rockville, MD). The K562 cell line was maintained in RPMI (Sigma-Aldrich, St. Louis, MO) supplemented with 10 % fetal bovine serum and 1 % penicillin-streptomycin (5 mg/mL penicillin, 5 mg/mL streptomycin; Thermo Fisher Scientific, Waltham, MA). The 293T cell line was maintained in DMEM (Sigma-Aldrich, St. Louis, MO) supplemented with 10 % fetal bovine serum. Cells were cultured in an incubator at 37 °C with humidified air and 5 % CO2. CD80 cDNA and CD83 cDNA were cloned by reverse transcription-polymerase chain reaction (RT-PCR) using cDNA from peripheral blood mononuclear cells (PBMCs). CD80 cDNA was cloned into the retroviral vector pMXs at the *Bam*HI and *Xho*I sites, and CD83 cDNA was cloned into pMXs at the *Hin*dIII and *Xho*I sites. The cDNA sequences were confirmed by DNA sequencing.

### 2.2 Establishment of aAPC-A24

Transduction of retroviral vectors were performed as described previously [11]. Briefly, retroviral transduction was carried out using supernatants from PLAT-A cells transfected with retroviral vectors via PEI-Max (Cosmo Bio. Inc., Tokyo, Japan). The transduced cells were analyzed by FACS Aria (BD Biosciences, Bedford, MA, USA) using APC-conjugated anti-CD80 antibody (clone: 2D10, BioLegend, San Diego, CA, USA), PE-conjugated anti-CD83 antibody (clone: HB15e, BioLegend), and anti-HLA-A24 antibody (clone: C7709A2.6, a kind gift from Dr. Pierre G. Coulie, Brussels, Belgium). CD80, CD83, and HLA-A24-positive cells were sorted and cultured, and the expression of CD80, CD83, and HLA-A24 was confirmed by flow cytometry. The expressions of CD80, CD83, HLA class I and β-Actin were also confirmed by Western blots. Anti-CD80 antibody (clone: E3Q9V, dilution Cell Signaling Technology, Danvers, MA, USA), anti-CD83 antibody (clone: D8V7V, Cell Signaling Technology), anti-HLA class I (clone: EMR8-5, Hokudo, Sapporo, Japan) and anti-β-Actin antibody (clone: AC-15, Sigma-Aldrich, Inc., St. Louis, MO, USA) were used. All primary antibodies were used at 1:1000 dilutions. The second antibodies were used at 1:5000 dilution. Western blots were performed as described previously [12].

### 2.3 SP-MITD gene design and construction and overexpression

**Fig. 1:**
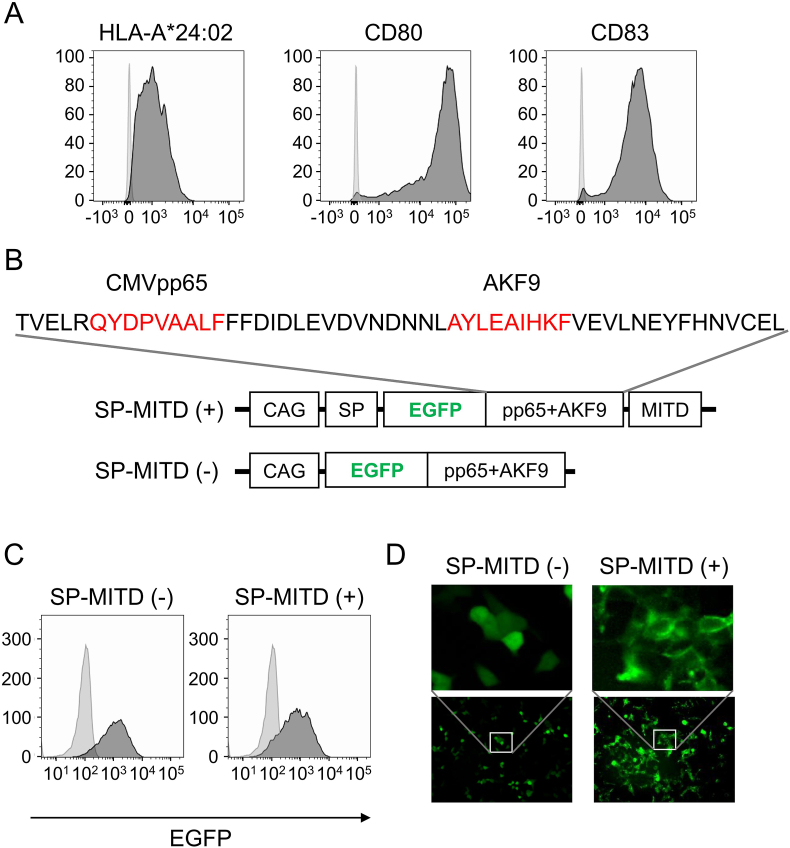
Establishment of aAPC-A24 and SP-MITD construct A. Expressions of HLA-A∗24:02, CD80 and CD83 K^56^2 cells were transduced with HLA-A∗24:02, CD80 and CD83 genes. Then, the expressions of HLA-A∗24:02, CD80 and CD83 were addressed by a flow cytometry using specific antibodies. B. Design of constructs EGFP gene were fused with epitope minigenes with franking sequence. For SP-MITD (+) construct, signal peptide (SP) sequence and MHC class I trafficking signal (MITD) sequences. C. Expression of EGFP in aAPC-A24 SP-MITD (−) gene and SP-MITD (+) gene were transduced into aAPC-A24, then, the expressions of EGFP were addressed by a flow cytometry. D. Fluorescent microscopySP-MITD (−) or SP-MITD (+) transduced 293T cells were observed by a fluorescent microscopy. Magnification, 200×.

SP-MITD (+) and SP-MITD (−) constructs were designed as shown in Fig. 1B. The genes were synthesized by GeneArt (Thermo Fisher Scientific) (the sequence are in supplemental Data 1) and cloned into pMXs-CAG at the *Bam*HI and *Xho*I sites. These genes were transduced into aAPC-A24 cells using retroviral transduction. EGFP-positive cells were isolated using FACS Aria and subsequently cultured. The expression of EGFP was confirmed by FACS Canto (BD Biosciences). For transfection into 293T cells, Lipofectamine 3000 (Invitrogen) was used. Fluorescent microscopy was performed using a BZ-X700 microscope (KEYENCE, Osaka, Japan).

### 2.4 CTL induction flow cytometry and IFNγ ELISPOT assay

The isolation of PBMCs, CTL induction, and establishment of CTL clones were performed as previously described with some modifications [13]. Peripheral blood mononuclear cells were isolated from HLA-A24-positive donors using Lymphoprep (Cosmo Bio Inc., Tokyo, Japan). CD8^+^ T cells were isolated using CD8 MicroBeads (Miltenyi Biotec, Tokyo, Japan) according to the manufacturer's protocol. The CD8^+^ T cells were seeded in 24-well plates (Corning Inc., Corning, NY) at a density of 2 × 10^6 cells/well in 1 mL of AIM-V medium (Thermo Fisher Scientific) supplemented with 10 % human serum (HS). The CD8^+^ T cells were stimulated weekly with peptide-pulsed aAPC-A24, SP-MITD (−) transduced aAPC-A24, or SP-MITD (+) transduced aAPC-A24. The aAPCs were irradiated with 100 Gy prior to use. Interleukin-2 (IL-2, 10 U/mL; PeproTech, Cranbury, NJ), IL-15 (10 ng/mL; PeproTech), and IL-21 (10 ng/mL; PeproTech) were added every 3 days. For peptide pulsing, CMVpp65 HLA-A24 epitope (QYDPVAALF) and AKF9 (AYLEAIHKF) peptides were incubated with aAPC-A24 at a concentration of 20 μg/mL for 2 h at room temperature, then washed three times before use for stimulation. The IFN-γ ELISPOT assay was performed as previously described [14].

### 2.5 Statistical analysis

Data were analyzed using one-way ANOVA followed by Dunnett's post hoc test, unless otherwise noted. All analyses were performed with GraphPad Prism (v9.5.0) (GraphPad, San Diego, CA). Results were considered significant at *P* < 0.05.

## 3 Results

### 3.1 Establishment of HLA-A∗24:02-positive artificial antigen presenting cells (aAPCs)

In this study, we aimed to optimize the efficacy of CTL induction in the context of HLA-A*24:02 using artificial antigen-presenting cells (aAPCs). First, we established HLA-A*24:02-positive aAPCs using the human erythroleukemia cell line K562. We transfected the cells with co-stimulatory molecules CD80 and CD83 and isolated CD80-positive cells. Subsequently, HLA-A*24:02* cDNA was stably transduced. The expressions of HLA-A24:02, CD80, and CD83 were confirmed by flow cytometry (Fig. 1A). The expressions of HLA-class I, CD80 and CD83 were also confirmed Western blots (Supplemental Data 2). These cells were designated as aAPC-A24.

### 3.2 Design of SP MITD construct and expression

We compared the cytotoxic T lymphocyte (CTL) induction potency of synthetic peptides versus endogenously expressed peptides. To achieve this, we designed synthetic epitope genes fused with EGFP, with and without SP-MITD [SP-MITD (+) and SP-MITD (−), respectively] (Fig. 1B). As epitopes, we used the HLA-A24-restricted CMVpp65 epitope as a representative viral antigen and the HLA-A24-restricted AKF9 epitope encoded by the mutated AP2S1 gene as a representative cancer antigen [15,16]. We incorporated a signal peptide (SP) and MHC class I trafficking signal (MITD) sequence into the synthetic epitope gene, as the SP-MITD sequence has been shown to significantly enhance antigen presentation efficacy when transduced into dendritic cells (DCs) [17]. In the SP-MITD (+) construct, SP was fused at the 5′ end of the EGFP/epitope, and the MITD sequence was fused at the 3’ end (Fig. 1B). The SP-MITD (−) construct lacked both the SP and MITD sequences (Fig. 1B). We then transiently transfected these plasmids into 293T cells. Cells transfected with SP-MITD (−) and SP-MITD (+) constructs expressed EGFP at equivalent levels (Fig. 1C). To determine the localization of EGFP, the cells were analyzed using fluorescence microscopy. In cells transfected with the SP-MITD (−) construct, EGFP exhibited a diffuse cytoplasmic distribution, while in cells transfected with the SP-MITD (+) construct, EGFP was partially localized on the cell surface (Fig. 1D). This result indicates that the SP and MITD sequences derived from HLA-A24 function properly.

### 3.3 CTL induction efficacy of aAPC transduced with SP-MITD (+)

**Fig. 2:**
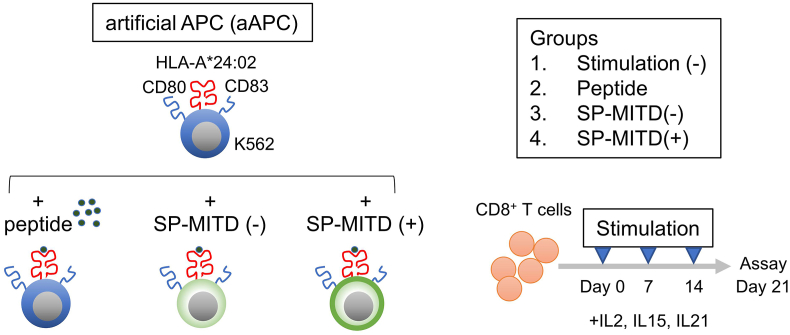
Schematic summary of CTL induction Antigenic peptide specific cytotoxic T lymphocytes (CTLs) were induced from peripheral blood mononuclear cells (PBMCs). Group 1: aAPC-A24 without antigens. Group 2: antigenic peptide pulsed-aAPC-A24. Group 3: SP-MITD (−) transduced aAPC-A24. Group 4: SP-MITD (+) transduced aAPC-A24. Each antigen presenting cell was used for stimulation of CD8^+^ T cells after irradiation. CD8^+^ T cells were stimulated weekly, then on Day21 the T cells were addressed for antigen specificity.

**Fig. 3:**
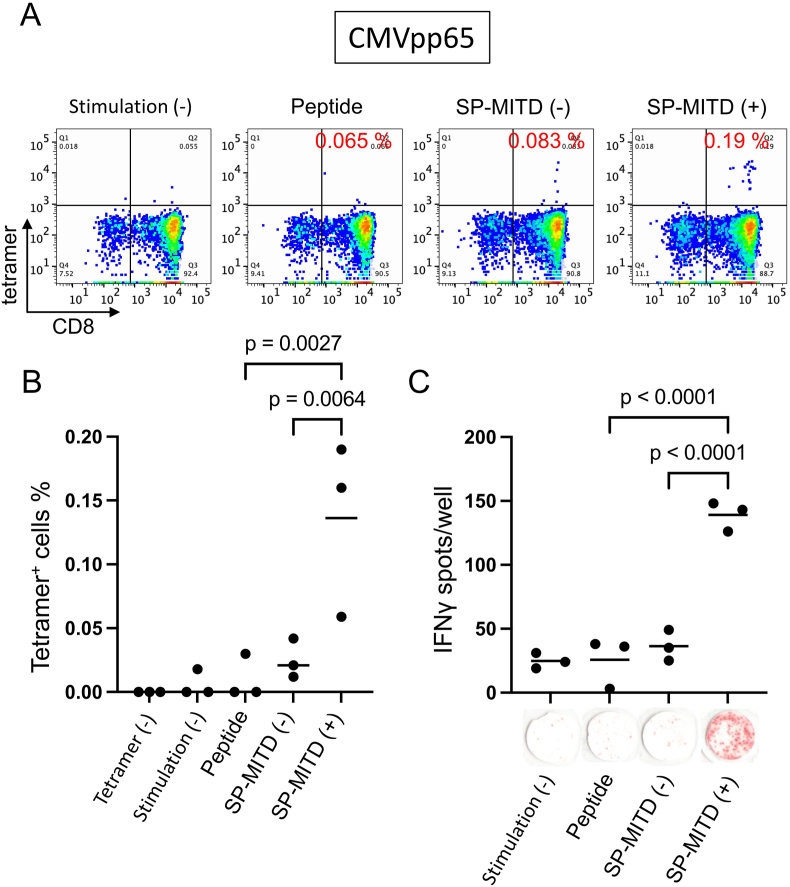
Induction of CTL specific for CMVpp65 using aAPC-A24 A, B. Flow cytometry of CTLs The CTLs were stained by CMVpp65 peptide and HLA-A24 complex tetramer and anti-human CD8 antibody, then analyzed by a flow cytometer (A). The gate for tetramer was defined by a sample without tetramer staining. The tetramer positive rates were analyzed in triplicate (B). C. IFNγ ELISPOT assay IFNγ ELISPOT assay was performed using peptide pulsed T2-A24 cells. The IFNγ-spots were counted in triplicate sample.

The CTL induction procedure is summarized in Fig. 2. CD8^+^ T cells were isolated from peripheral blood mononuclear cells (PBMCs) and then stimulated using aAPCs with three different antigen types: peptide-pulsed aAPCs, SP-MITD (−) transduced aAPCs, and SP-MITD (+) transduced aAPCs (Fig. 2). The induction of CMVpp65 epitope-specific CTLs was assessed by flow cytometry using the CMVpp65 peptide-HLA-A∗24:02 complex tetramer. The tetramer-positive rates for peptide-pulsed aAPCs, SP-MITD (−) aAPCs, and SP-MITD (+) aAPCs were 0.065 %, 0.083 %, and 0.19 %, respectively (Fig. 3A), with statistically significant differences among triplicate samples (Fig. 3B). Additionally, CTL function was evaluated using an IFN-γ ELISPOT assay. The assay showed that CTLs induced with SP-MITD (+) aAPCs had significantly higher IFN-γ spot counts compared to those induced with peptide-pulsed aAPCs and SP-MITD (−) aAPCs (Fig. 3C).

**Fig. 4:**
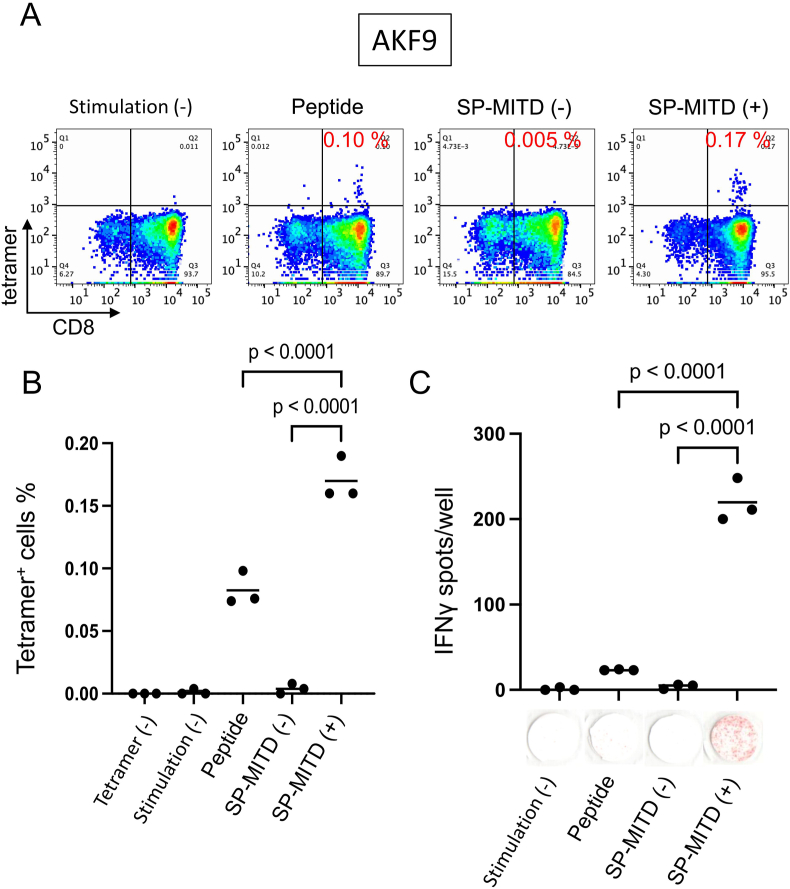
Induction of CTL specific for neoantigen AKF9 using aAPC-A24 A, B. Flow cytometry of CTLs The CTLs were stained by AKF9 peptide and HLA-A24 complex tetramer and anti-human CD8 antibody, then analyzed by a flow cytometer (A). The gate for tetramer was defined by a sample without tetramer staining. The tetramer positive rates were analyzed in triplicate (B). C. IFNγ ELISPOT assay IFNγ ELISPOT assay was performed using peptide pulsed T2-A24 cells. The IFNγ-spots were counted in triplicate sample.

To further validate the effectiveness of SP-MITD (+) aAPCs, we assessed CTL induction using AKF9, a neoantigen model. The tetramer-positive rates for peptide-pulsed aAPCs, SP-MITD (−) aAPCs, and SP-MITD (+) aAPCs were 0.1 %, 0.005 %, and 0.17 %, respectively (Fig. 4A), with statistically significant differences among triplicate samples (Fig. 4B). The IFN-γ ELISPOT assay also demonstrated that SP-MITD (+) aAPCs induced significantly more IFN-γ spots compared to peptide-pulsed aAPCs and SP-MITD (−) aAPCs (Fig. 4C).

## 4 Discussion

In this study, we demonstrated that SP-MITD (+) aAPCs exhibit superior CTL induction compared to peptide-pulsed aAPCs. Although we used a high concentration of synthetic peptide (20 μg/mL), which saturated the exogenously pulsed peptide, the CTL induction efficiency of peptide-pulsed aAPCs was still lower than that of SP-MITD (+) aAPCs. Possible mechanisms for this include (1) peptide exchange rate and (2) continuous presentation of endogenous peptides. K562-based aAPCs express antigen presentation machinery (APM) molecules, which should allow for the expression of endogenously processed peptides. While exogenously pulsed peptides can bind to empty HLA class I molecules, they may also be taken up and presented on HLA class I molecules [18]. Therefore, exogenously pulsed peptides might be less effective than peptides overexpressed by the SP-MITD (+) construct. Another possible explanation is the continuous presentation of endogenous peptides. The antigenic peptides derived from the SP-MITD (+) construct are continuously presented during CTL stimulation, whereas peptide-pulsed aAPCs may decrease in antigenic peptide availability during the stimulation process.

CTL induction efficiency with the SP-MITD (+) construct was superior to that of the SP-MITD (−) construct. In fact, very few CTLs could be detected in the SP-MITD (−) construct (Fig. 3, Fig. 4). In L929 cells, dendritic cells, and macrophages, a single antigenic peptide can be produced from 500 to 3000 viral product degradations [19]. To improve the efficiency of antigenic peptide production from mature proteins, Kreiter et al. introduced the SP-MITD sequence for CTL induction using DCs [17]. Although the extent to which SP-MITD improves antigenic peptide production is not fully known, it has been shown to enhance CD8^+^ and CD4^+^ T cell stimulation. In this study, we introduced SP-MITD into K562-based aAPCs for the first time and found that it significantly improved CTL induction. Thus, T cell activation using aAPCs can be enhanced by SP-MITD. In this study, IL-2, IL-15 and IL-21 were used for CTL induction. IL-2 promotes T cell proliferation, IL-15 sustains memory T cells, and IL-21 enhances effector functions of CTLs [20].

aAPCs are used for both specific and non-specific T cell activation [7]. DCs are known as professional APCs because of the powerful antigen presenting capability to CTLs [4]. DCs can be generated from human monocytes derived from PBMCs using certain cytokines [21]; however, the cost of cytokines are concerns. On the other hand, aAPCs derived from K562 cells can be produced in large quantities with consistent quality at low cost. Thus, aAPCs can provide versatile platform to generate CTLs. Antigenic peptide-pulsed aAPCs can induce peptide-specific T cell responses [22]. Additionally, aAPCs transduced with antigen mini-genes fused with proteasomal cleavage sequences have demonstrated better CTL induction efficiency compared to peptide-pulsed aAPCs [23]. Thus, naturally processed and endogenously presented antigenic peptides are efficient in stimulating CTLs. However, the comparative efficacy of SP-MITD (+) transduced aAPCs and mini-gene-fused proteasomal cleavage site aAPCs is still not fully understood. The advantage of the SP-MITD (+) system is its potential for identifying unknown epitopes. Screening for neoantigens encoded by mutated genes is challenging. Although prediction of HLA binding is highly accurate [24,25], validation through CTL induction remains essential. The use of tandem mini-genes (TMGs) fused with SP-MITD may be an effective approach for epitope screening in neoantigens.

In summary, our study highlights that SP-MITD (+) transduced aAPCs provide the highest CTL induction efficacy compared to peptide-pulsed aAPCs and SP-MITD (−) transduced aAPCs. This system is valuable for further CTL induction experiments.

## CRediT authorship contribution statement

**Kenta Sasaki:** Writing – original draft, Methodology, Investigation, Data curation, Conceptualization. **Kenji Murata:** Methodology, Formal analysis, Data curation. **Tomoyuki Minowa:** Methodology, Formal analysis, Data curation. **Naoki Shijubou:** Data curation. **Hiroki Kobayashi:** Data curation. **Munehide Nakatsugawa:** Conceptualization. **Aiko Murai:** Data curation. **Yuka Mizue:** Data curation. **Terufumi Kubo:** Formal analysis. **Takayuki Kanaseki:** Formal analysis, Data curation, Conceptualization. **Tomohide Tsukahara:** Formal analysis. **Hisashi Uhara:** Supervision. **Akemi Ishida-Yamamoto:** Supervision. **Yoshihiko Hirohashi:** Writing – review & editing, Writing – original draft, Supervision, Methodology, Investigation, Funding acquisition, Formal analysis, Conceptualization. **Toshihiko Torigoe:** Writing – original draft, Validation, Supervision, Formal analysis, Conceptualization.

## Declaration of competing interest

The authors declare that they have no known competing financial interests or personal relationships that could have appeared to influence the work reported in this paper.

## Figures and Tables

**figs1:**