# Supplementary material for: MHC class I trafficking signal improves induction of cytotoxic T lymphocyte using artificial antigen presenting cells
Source: Biochem Biophys Rep. 2025 Feb 19;41:101946. doi: 10.1016/j.bbrep.2025.101946 (PMC11880709; doi:10.1016/j.bbrep.2025.101946)
Supplement: Multimedia component 1 [file mmc1.docx]

Supplemental Data 1.

The sequence of SP-MITD (+) and SP-MITD (-) constructs.

SP-MITD(+)

GCCACCATGGCCGTGATGGCCCCTAGAACACTGGTGCTGTTGCTGTCTGGTGCCCTGGCTCTGACACAGACATGGGCCGGCTCTGTGTCCAAGGGCGAAGAACTGTTTACCGGCGTGGTGCCCATCCTGGTGGAACTGGATGGGGATGTGAACGGCCACAAGTTCAGCGTTAGCGGAGAAGGCGAAGGCGACGCCACATACGGAAAGCTGACCCTGAAGTTCATCTGCACCACCGGCAAGCTGCCTGTGCCTTGGCCTACACTGGTCACCACACTGACATACGGCGTGCAGTGCTTCAGCAGATACCCCGACCATATGAAGCAGCACGACTTCTTCAAGAGCGCCATGCCTGAGGGCTACGTGCAAGAGCGGACCATCTTCTTTAAGGACGACGGCAACTACAAGACCAGGGCCGAAGTGAAGTTCGAGGGCGACACCCTGGTCAACCGGATCGAGCTGAAGGGCATCGACTTCAAAGAGGACGGCAACATCCTGGGCCACAAGCTTGAGTACAACTACAACAGCCACAACGTGTACATCATGGCCGACAAGCAGAAAAACGGCATCAAAGTGAACTTCAAGATCCGGCACAACATCGAGGACGGCTCAGTGCAGCTGGCCGATCACTACCAGCAGAACACACCCATCGGAGATGGCCCTGTGCTGCTGCCCGATAACCACTACCTGAGCACACAGAGCGCCCTGAGCAAGGACCCCAACGAGAAGAGGGATCACATGGTGCTGCTGGAATTCGTGACCGCCGCTGGCATCACACTCGGCATGGATGAGCTGTACAAACCGTGGAACTGCGTCAGTACGATCCCGTGGCTGCGCTCTTCTTTTTCGATATCGACCTCGAGGTGGACGTGAACGACAACAACCTGGCCTACCTGGAAGCCATCCACAAGTTTGTGGAAGTGCTGAACGAGTACTTCCATAACGTGTGCGAGCTGATCATTGCCGGCCTGGTTCTGCTCGGAGCCGTGATTACAGGTGCTGTGGTGGCTGCTGTGATGTGGCGGAGAAACAGCAGCGACAGAAAAGGCGGCAGCTACTCTCAGGCCGCCAGCTCTGATTCTGCCCAGGGCTCTGATGTGTCTCTGACCGCCTGCAAGGTGTAG

SP-MITD(-)

GCCACCATGGTGTCCAAGGGCGAAGAACTGTTTACCGGCGTGGTGCCCATCCTGGTGGAACTGGATGGGGATGTGAACGGCCACAAGTTCAGCGTTAGCGGAGAAGGCGAAGGCGACGCCACATACGGAAAGCTGACCCTGAAGTTCATCTGCACCACCGGCAAGCTGCCTGTGCCTTGGCCTACACTGGTCACCACACTGACATACGGCGTGCAGTGCTTCAGCAGATACCCCGACCATATGAAGCAGCACGACTTCTTCAAGAGCGCCATGCCTGAGGGCTACGTGCAAGAGCGGACCATCTTCTTTAAGGACGACGGCAACTACAAGACCAGGGCCGAAGTGAAGTTCGAGGGCGACACCCTGGTCAACCGGATCGAGCTGAAGGGCATCGACTTCAAAGAGGACGGCAACATCCTGGGCCACAAGCTTGAGTACAACTACAACAGCCACAACGTGTACATCATGGCCGACAAGCAGAAAAACGGCATCAAAGTGAACTTCAAGATCCGGCACAACATCGAGGACGGCTCAGTGCAGCTGGCCGATCACTACCAGCAGAACACACCCATCGGAGATGGCCCTGTGCTGCTGCCCGATAACCACTACCTGAGCACACAGAGCGCCCTGAGCAAGGACCCCAACGAGAAGAGGGATCACATGGTGCTGCTGGAATTCGTGACCGCCGCTGGCATCACACTCGGCATGGATGAGCTGTACAAACCGTGGAACTGCGTCAGTACGATCCCGTGGCTGCGCTCTTCTTTTTCGATATCGACCTCGAGGTGGACGTGAACGACAACAACCTGGCCTACCTGGAAGCCATCCACAAGTTTGTGGAAGTGCTGAACGAGTACTTCCATAACGTGTGCGAGCTGTAG

Blue: SP-MITD

Green: EGFP

Yellow: CMV pp65

Red: AKF9
